# Eco-anxiety, Psychology, and Theological Beliefs About Creation Among Clergy and Lay People in the Church of England

**DOI:** 10.1007/s10943-026-02760-w

**Published:** 2026-09-12

**Authors:** Andrew Village, Leslie J. Francis

**Affiliations:** 1 York St John University, York, UK; 2 Centre for Research in Intellectual and Developmental Disabilities (CIDD), University of Warwick, Coventry, UK; 3 World Religions and Education Research Unit, Lincoln Bishop University, Lincoln, UK

**Keywords:** Church of England, Dominion, Eco-anxiety, Eschatology, Evangelicals, Pro-environmental behaviour, Psychological type, Stewardship

## Abstract

Interest in eco-anxiety is growing rapidly, partly for therapeutic reasons and partly because it may be a motivation for pro-environmental behaviours. There is little study of how religious identities or church traditions and specific theological beliefs are related to eco-anxiety. This cross-sectional survey of 3240 clergy and laity from the Church of England explored the relationships between eco-anxiety, psychological dispositions, church tradition, and specific theological beliefs about creation. Eco-anxiety was measured using a subset of items from the Hogg Eco-Anxiety Scale. The number of eco-friendly activities increased with eco-anxiety, although the relationship was asymptotic rather than linear. The distribution of eco-anxiety symptom counts was zero inflated. Those recording zero symptoms were more likely to be younger, male, and Evangelical than those recording one or more symptoms. Recording zero symptoms seemed to be partly a mark of ideological ‘eco-denial’. Psychological predictors of eco-anxiety were high emotional volatility scores (neuroticism), low thinking scores, and, to a lesser extent, low sensing scores. Theological beliefs about human dominion over creation and about divine rather than human control of the fate of the Earth predicted lower eco-anxiety, while theological beliefs about human stewardship and about the sacramental nature of creation predicted higher eco-anxiety. The effect of theological conservatism on predicting zero eco-anxiety was less important for Evangelicals, who were generally more conservative, than it was for other church traditions. Eco-anxiety in this religious sample was shaped by the complex effects of psychological dispositions, specific theological beliefs about creation, church tradition, and theological conservatism.

## Introduction

Eco-anxiety is a term applied to a range of different psychological responses to real or perceived environmental crises. Symptoms associated with eco-anxiety include physical responses (sleeplessness, being sick, or having panic attacks) and negative emotional responses (irritability, weakness, sadness, depression, numbness, helplessness, hopelessness, guilt, frustration, and anger) (Coffey et al., 2021). Kurth and Pihkala (2022) point out that researchers have used the term ‘anxiety’ loosely to refer to different affective responses that may be anxiety related (nervousness or fear), self-reflective (shame or guilt), or grief-related (being upset or distressed). However, it is defined, eco-anxiety is generally perceived as being a low-level chronic response in most people (Pihkala, 2020).

Interest in eco-anxiety is partly about its effects on mental health (Albrecht, 2011; Clayton, 2020; Innocenti et al., 2025; Wu et al., 2020) and partly about its relation to concern over the environment and motivation for more eco-friendly attitudes and pro-environmental behaviours. Some surveys have shown a positive correlation between eco-anxiety and pro-environmental behaviour (Jain & Jain, 2022; Kricorian et al., 2025; Pavani et al., 2023; Verplanken et al., 2020; Whitmarsh et al., 2022), whereas others have found little relationship (Stanley et al., 2021). This may be because different responses associated with ‘eco-anxiety’ operate differently in motivating action (Hogg et al., 2024; Merino et al., 2025). It has been suggested that, while low levels of eco-anxiety may promote action, excessive eco-anxiety could have the opposite effect (Wullenkord et al., 2021), but there is little evidence to support this suggestion (Hogg et al., 2024). Although eco-anxiety can be construed as a largely negative response to environmental issues, it may also be a marker of environmental awareness and motivation to individual or corporate action to address the problem. Some low-level anxiety (or related negative affect) may be a healthy response to perceived threat, so total lack of anxiety may point to a more deliberate choice to downplay what is widely regarded as one of the more serious threats of our time.

As well as being associated with direct experience of ecological crises, eco-anxiety may also vary between individuals for sociodemographic reasons. Women tend to show greater eco-anxiety than men (Niedzwiedz & Katikireddi, 2023; Regener, 2025). A lot of attention has been focussed on children and young adults because they may bear the brunt of climate change. The evidence for age-related differences across all adults in eco-anxiety is currently unclear: the European Social Survey in 2020–2022 showed those in their 60s were more anxious than teenagers (Niedzwiedz & Katikireddi, 2023), but a recent study in Italy found that those aged 18–28 were four times more likely to report high eco-anxiety compared to those aged 60–78 (Marcolini et al., 2025). Eco-anxiety seems to be partly related to trait measures of negative affect such as neuroticism (Hempel et al., 2026; Ogunbode et al., 2024; Pavani et al., 2023), although evidence linking other personality traits is limited.

Eco-anxiety and concern for the environment have also been shown to be related to religion, although the complexity of religion and differences between denominational and individual-level attitudes means that there is no simple link between religious affiliations, religious beliefs, attitudes towards the environment, and eco-anxiety. The effects of religion on environmental attitudes vary both between and within religions (Arbuckle, 2017; Ferber & Haluza-DeLay, 2015; Morrison et al., 2015; Toros, 2025), so detailed studies within specific religious groups may offer more nuanced understandings of the relationship of eco-anxiety to religion. This paper examines eco-anxiety among clergy and lay people belonging to the Church of England, which is part of the wider Anglican Communion. The aim was to explore the how psychological dispositions, religious identities, church traditions, and specific theological beliefs about creation shape levels of eco-anxiety in this religious group. Understanding the traditions in the Church of England and their psychological profiles is therefore important for the purposes of this study.

### Religious Identity and Church Traditions in the Church of England

The Church of England is a national Church that is influenced both by its Catholic roots and by the Protestant Reformation, two distinct church traditions that are still present in the two main wings of the Church, the Anglo-Catholic (Hylson-Smith, 1993) and Evangelical (Hylson-Smith, 1989). Alongside these have been the development of liberalism, which came to prominence in the nineteenth century and which tended to influence the Catholic rather than the Evangelical wing of the Church (Duggan, 2022; Ward, 1964). Empirical studies of the present-day Church of England have shown that two correlated polarities are the best measures of identity and expression of faith within the denomination: Anglo-Catholic versus Evangelical, and Liberal versus Conservative. Two seven-point bipolar scales have been used in surveys to measure these two aspects of faith expression (Randall, 2005; Village, 2012b, 2018a, 2018b). In general, those who rate themselves towards the Evangelical end of the scale tend to be more conservative on matters of doctrine and morality and to stress the authority of the Bible over the Church. Those who rate themselves towards the Anglo-Catholic end of the scale tend to be more liberal in doctrine and morality, although extreme Anglo-Catholics tend to be slightly more conservative, especially on issues such as the ordination of women. Those who locate themselves in the middle of the scale (scoring 3–5) are termed ‘Broad Church’ and demonstrate views that often fall between the two polarities (Village, 2012b).

These two scales have been tested on large samples of clergy and lay people from the Church of England in three successive surveys in 2001 (Francis et al., 2005), 2013 (Village, 2018a), and the present study. In each case, the correlation of the scales showed a similar ‘hockey stick’ shape, reflecting the conservatism of Evangelicals, with extreme Anglo-Catholics being slightly more conservative that the Broad Church but not as conservative as Evangelicals (see Appendix, Fig. 3).

### Psychology and Church Tradition in the Church of England

The psychology of clergy and lay people in the Church of England has been widely studied since the 1990s using the psychological-type model created by Carl Jung (1923) and subsequently developed by others (Francis, 2005; Francis & Village, 2022; Myers, 1993; Myers & Myers, 1980). The two main strands of this work have been first to use the typology to explore the profiles of different groups (Francis & Lankshear, 2021; Francis et al., 2011, 2012, 2019; Muskett & Village, 2015; Village, 2023) and second to explore the beliefs and attitudes that are associated with different type preferences (Village & Francis, 2005; Village, 2012a, 2016, 2019).

The two core processes of the psychological-type model are the perceiving process and the judging process, both of which have been shown to be important in shaping religious identities and faith expression. The perceiving process is conceptualised as the way in which information is gathered, which can be through the functions of sensing or intuition. Those who prefer sensing over intuition tend to be down-to earth, practical, and wedded to the familiar; those who prefer intuition over sensing tend to be open to new possibilities, to linking ideas in novel ways, and to creative thinking. This process is parallel to the dimension of openness in the big five factor model, with high scores in intuition being associated with high scores in openness (McCrae & Costa, 1989). The difference is that in the type model, those who score low on openness (intuition) would not be considered as closed-minded or deficient in some way but instead would be seen as scoring high on sensing, a different but equally valid way of approaching perception.

The judging process is conceptualised as the way in which decisions are made, which can be through the functions of feeling or thinking. Those who prefer feeling over thinking tend to have a strong sense of empathy with others, to prefer to reach consensus, and to draw on shared values when deciding on matters. Those who prefer thinking over feeling tend to draw on logic and evidence to make decisions and to be guided by principles, even if that might risk upsetting others. This process is parallel to the dimension of agreeableness in the big five factor model, with high scores in feeling being associated with high scores in agreeableness (McCrae & Costa, 1989). The difference is that in the type model, those with low agreeableness (feeling) would not be considered as disagreeable or deficient in some way but instead would be seen as scoring high on thinking, a different but equally valid way of making decisions.

Within the Church of England, psychological type is associated with church tradition and theological beliefs. Among both clergy and laity, Anglo-Catholics tend to score higher on intuition and on feeling than do Evangelicals (Francis et al., 2020; Village, 2013; Village et al., 2009). Preference for sensing over intuition is associated with biblical conservatism (Village, 2016) and biblical literalism (Village, 2012a), which is also seen in samples that include other denominations (Village, 2014). Scores on the liberal versus conservative scale are also related to psychological-type preferences, with preference for thinking being associated with conservatism rather than liberalism (Village, 2019).

### Religion and Environmentalism

Within Christianity, both denominational affiliations or church traditions and specific theological beliefs have been shown to be related to environmentalism, including eco- or climate anxiety. Much of this work has been developed in the USA, where Lynn White’s seminal paper argued that it was literal interpretation of the Genesis account of creation, with the mandate for humans to have dominion over the created order, that has been the root cause of what he termed the ‘ecologic crisis’ (White, 1967). Work since then has shown contradictory results (see Sherkat & Ellison, 2007), but the generally lower support for the environment by Evangelical Protestants has been widely documented (see Schwadel & Johnson, 2017). This is partly connected to wider support for Republicanism but mainly related to specific theological beliefs and interpretation of the bible. Several studies have suggested that it not just beliefs about the creation narratives that shape attitudes in this group, but also beliefs about ‘end times’ (eschatology). In particular, the sense that there is divine control over the fate of the Earth tends to reduce concerns over the environment and willingness to support actions to protect it (Barker & Bearce, 2013; Guth et al., 1995; Sherkat & Ellison, 2007).

Two studies on churchgoers in the UK have examined in detail the relationship between specific theological beliefs and concern for the environment. The first study by Village (2015) examined the effects of biblical literalism and theological beliefs about creation on concern for the environment among a sample of 537 churchgoers from a range of mainly Protestant denominations. The study developed scales measuring three different theological stances towards creation: dominion (humans have a mandate to use creation for their own good), sacramental (creation is the prime source of divine revelation), and stewardship (humans have a responsibility to God for caring for creation). Biblical literalism had both a direct negative correlation with environmental concern, and indirect effects through promoting belief in both dominion and stewardship. The latter was an important stance that was positively correlated with concern for the environment. The second study by Village (2020) among a sample of 825 churchgoers expanded this analysis to include psychological variables and an additional measure of eschatology (divine rather than human control of the fate of the Earth). Psychological-type variables had indirect negative effects on environmental concern: sensing through promoting literalism, which was associated with dominion and eschatology, and thinking through positive association with belonging to Evangelical Protestant denominations and theological conservatism, which in turn were associated with literalism, dominion, and eschatology. Emotional volatility (a measure of trait neuroticism) was directly positively correlated with environmental concern, but not with theological beliefs.

### Research Questions

The evidence suggests that eco-anxiety in the Church of England could be driven by a range of factors that might produce direct and indirect effects. Psychological variables such as neuroticism may directly influence eco-anxiety because individuals who are generally emotionally volatile are likely to be susceptible to anxieties with a specific cause. Psychological-type dispositions may be directly related to eco-anxiety, but also indirectly if they are also associated with church tradition, levels of conservatism, or specific theological beliefs about creation. Specific theological beliefs about the relationship of humans to creation may have direct effects in increasing or reducing eco-anxiety. Those who believe in a divine mandate for human dominion over creation, or who believe the fate of the Earth is under divine control may be less anxious about the environment. Those who have a sacramental understanding of creation, or who believe that humans are God’s stewards of creation may be anxious that humans seem to be doing a bad job of caring for the Earth. Adherence to particular church traditions, or general liberal or conservative faith stance, may also shape views about the environment that might for some make eco-anxiety appear as an ideological creation of the liberal left. This effect may operate through theological beliefs about creation, but might also foster a denial of eco-crisis and therefore a denial of eco-anxiety as a possibility. In this case, some individuals might register no evidence of eco-anxiety not because they are psychologically unlikely to suffer, but because of ideological ‘eco-denial’.

Against this background, this paper explores three main questions among a sample of committed Christians. Among this sample:

1. What is the relationship of eco-anxiety to eco-activity? Might low levels of anxiety increase ecological friendly behaviours, and is there any evidence that high levels may attenuate or decrease such behaviours?
2. Is there any evidence of ‘eco-denial’ in the frequency distribution of eco-anxiety and, if so, which people in the sample are most likely to fall into this category?
3. How do levels of eco-anxiety relate to religious identity and church tradition, psychological dispositions, and specific theological beliefs about creation?

## Method

### Procedure and Participants

The *Church 2024* survey was an online survey delivered using the Qualtrics platform and available from March to November 2024. Invitations to participate were published during the year in the main Church of England newspapers, the *Church Times* and the *Church of England Newspaper*, as well as in a number of diocesan newsletters. Of the 5141 total responses to the survey, 3826 (74.4%) were people living in England who affiliated with the Church of England. The final sample used here consisted of 3240 individuals who had no missing values for variables used in this analysis (Table 1). Comparison of the sample with available data from the Church of England suggests, as far as the available national data allow, that the sample was reasonably representation of the Church of England as whole (see Village, 2025 for more details).

**Table 1:** Profile of the sample

		%
Sex	Male	48.3
	Female	51.7
Age	20s	4.0
	30s	7.7
	40s	13.1
	50s	21.4
	60s	26.2
	70s	21.6
	80s+	5.9
Ethnicity	Black and Minority Ethnic	5.8
	White British	94.2
Tradition	Anglo-Catholic	28.3
	Broad Church	44.5
	Evangelical	27.1
Status	Lay	60.2
	Clergy	39.8

*Note*: *N* = 3240

The survey was intended to assess a wide range of attitudes and beliefs, so it was important to minimise the number of items to reduce completion times and increase completion rates. A section on attitudes towards the environment included items designed to assess eco-concern, eco-activity, eco-anxiety, and a range of theological beliefs about the environment framed as divinely created. Item responses and properties for scales used in this analysis are shown in the Appendix. All scales achieved satisfactory to excellent internal consistency reliability as measured by Cronbach’s alpha (Cronbach, 1951) or McDonald’s omega (Hayes & Coutts, 2020).

### Environmental Variables

*Eco-anxiety* was assessed using a scale based on the first ten items of the 13-item Hogg Eco-Anxiety Scale (Hogg et al., 2021, 2023). This included the subscales assessing affective symptoms, rumination, and behavioural symptoms, but excluded anxiety about personal impact (Appendix, Table 5). Items were introduced using the rubric ‘This question is about how bothered you are by the following problems when you are thinking about climate change or other issues such as habitat loss, species extinction, pollution, waste management, or deforestation. When you are thinking about *environmental issues*, how often do you?’ Each item was rated on a four-point scale: never (0), sometimes (1), most of the time (2), constantly (3). For analysis, the four points were collapsed to a two-point scale: never (0), at least sometimes (1). This converted the eco-anxiety scale into a count of the number of individual items that had a nonzero response (i.e. the number of different symptoms recorded) and ranged from 0 to 10.

*Environmental activity* was assessed by a seven-item scale (‘Activity’) expanded from Village (2020) and introduced by the rubric ‘Have you ever?’ followed by items such as ‘changed what you eat for environmental reasons?’ or ‘actively protested about an environmental issue?’. Each item was rated on a two-point scale (no = 0, yes = 1). These items were used to create a scale of eco-friendly behaviour ranging from 0 to 7 (Appendix, Table 6).

**Table 2:** Profiles of individuals with zero eco-anxiety symptoms compared to those with one or more symptoms

		Eco-anxiety symptom count				
		Zero	1+			
	*N* =	320	2920			
Contingency tests		%	%	χ^2^	*df*	
Sex	Male	71.3	45.8			
	Female	28.7	54.2	74.9	1	***
Age	<40	23.1	10.4	39.7		
	40s	13.4	13.1			
	50s	23.8	21.1			
	60+	55.3	54.0	3	***	
Education	No degree	19.1	17.4			
	UG degree	37.5	41.8			
	PG degree	43.4	40.8	2.3	2	NS
Church tradition	Anglo-Catholic	27.2	28.5			
	Broad Church	31.3	46.0			
	Evangelical	41.6	25.5	41.5	2	***

		Eco-anxiety symptom count				
		Zero	1+			
	*N* =	320	2920			
*t*-tests		Mean (SE)	Mean (SE)	*t*	*df*	
	Neuroticism	1.30 (.097)	2.37 (.038)	− 10.3	3238	***
	Sensing	3.67 (.096)	3.45 (.032)	2.2	3238	*
	Thinking	4.29 (.102)	3.08 (.038)	11.1	3238	***

*Note*: *p < .05; ***p < .001

NS: Not significant

### Predictor and Control Variables

#### Personal Factors

Sex was coded male (0) and female (1) and used as a dummy variable in regressions labelled ‘female’. Age was coded: 18–19 (1), 20s (2), 30s (3), 40s (4), 50s (5), 60s (6), 70s (7), and 80s+ (8). The highest level of education was coded: no school qualifications (1), school-level (2), university certificate or diploma (3), undergraduate degree (4), masters degree (5), doctoral degree (6). Both age and highest level of education were treated as continuous variables in regressions.

#### Psychological Factors

Psychological variables were assessed using the revised shortened version of the Francis Psychological Type and Emotional Temperament Scales (FPTETS; Village & Francis, 2023a, 2023b, 2024)*.* This is a 30-item instrument comprising four sets of six forced-choice items related to each of the four components of psychological-type theory: orientation (extraversion or introversion), perceiving process (sensing or intuition), judging process (thinking or feeling), and attitude towards the outer world (judging or perceiving), and six items related to emotional temperament (calm or volatile), a measure of trait neuroticism (Village & Francis, 2022). Scores in each component were complementary and summed to six. Based on previous work on environmentalism using this instrument (Village, 2020) the scores for sensing, thinking, and emotional volatility (neuroticism) were used in regressions. Alpha reliabilities were .68 for the SN scale, .79 for the TF scale, and .82 for the emotional volatility scale.

#### Religious Identity and Church Tradition

Religious identity was assessed using a seven-point bipolar scale labelled ‘Anglo-Catholic’ at one end and ‘Evangelical’ at the other. It is a good indicator of differences in belief and practice in the Church of England (Randall, 2005; Village, 2012b, 2018a) and was used to identify Anglo-Catholic (scoring 1–2), Broad Church (3–5), and Evangelical (6–7) respondents. Two dummy variables, Anglo-Catholic and Evangelical, were used in the regression analyses. Religious identity, conceptualised as differentiating among Anglo-Catholic, Broad Church, and Evangelical, needs to be further nuanced by differentiating between conservative and liberal church traditions. The survey also included a seven-point bipolar scale used in other studies of the Church of England measuring general conservative (versus liberal) stance, the so-called ‘LIBCON’ scale (Randall, 2005; Village, 2018b). High scores indicate conservatism (and low scores liberalism) in matters of doctrine and morality. In the Church of England, Evangelicals tend to be more conservative than those in the Broad Church or Anglo-Catholic traditions. Those on the extreme wing of the Anglo-Catholic tradition tend to be slightly more conservative than those in the Broad Church tradition, but less conservative than Evangelicals (Appendix, Fig. 3).

**Fig. 1:**
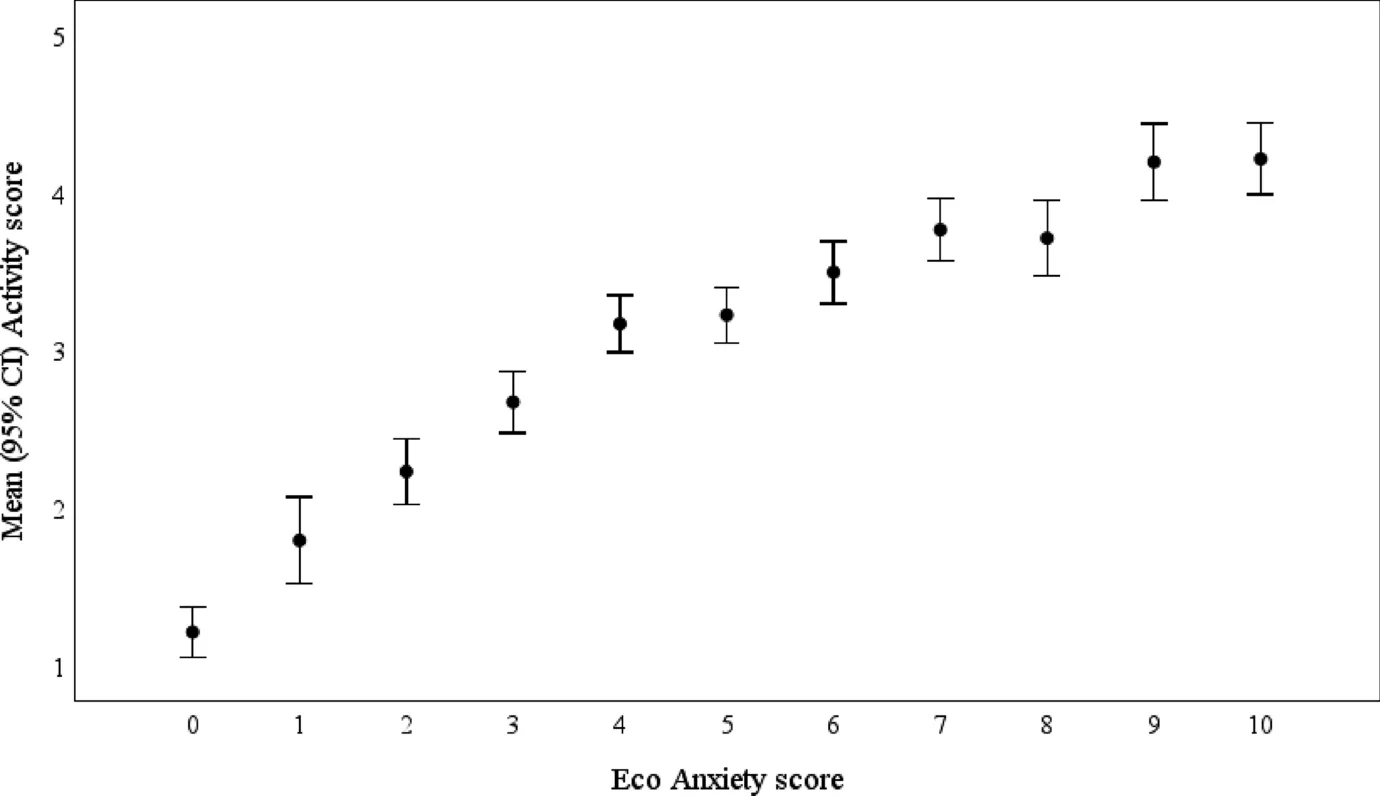
Eco-friendly activity score in relation to eco-anxiety score

#### Theological Beliefs About Creation

Four scales were based on those developed in previous studies of churchgoers from a range of denominations in the UK, including the Church of England (Village, 2015, 2020). They assessed four different theological stances towards creation (Appendix, Table A3). *Dominion* was assessed by a six-item scale coded such that high scores indicated belief in the superiority of humans over other living things and the right of humans to rule, dominate, or exploit the Earth. *Eschatology* was assessed by a six-item scale scored such that high scores indicated belief that God (rather than humans) controlled the fate of creation. *Stewardship* was assessed by a six-item scale coded such that high scores indicated belief that humans have a responsibility to God to care for the environment and preserve the Earth. *Sacramental* was assessed by a six-item scale coded such that high scores indicated belief that God is present in creation and the intimate connection of humans to all living things. Each item was rated on a five-point scale: disagree strongly (1), disagree (2), not certain (3), agree (4), agree strongly (5).

**Table 3:** Multiple regression of eco-anxiety scale count of symptoms

	Model 1	Model 2	Model 3	Model 4	Model 5	Model 6
Evangelical	− 0.209***	− 0.185***	− 0.145***	− 0.018	− 0.001	0.032
Anglo-Catholic	− 0.011	0.010	0.001	− 0.032	− 0.035	− 0.034
Female		0.150***	0.072***	0.040*	0.035*	0.035*
Age		0.012*	0.033***	0.018**	0.018**	0.018**
Education		− 0.016*	− 0.007	− 0.019*	− 0.020*	− 0.020*
Neuroticism			0.065***	0.059***	0.059***	0.059***
Thinking			− 0.030***	− 0.019***	− 0.018***	− 0.018***
Sensing			− 0.010*	− 0.007	− 0.005	− 0.005
Dominion				− 0.011***	− 0.009***	− 0.009***
Eschatology				− 0.020***	− 0.017***	− 0.017***
Stewardship				0.013***	0.014***	0.014***
Sacramental				0.018***	0.016***	0.016***
Conservatism					− 0.020***	− 0.018**
EV x Con						− 0.007

*Note*: *N* = 3240

Poisson distribution with log link. Estimates are the predicted changes in log symptom count for a one-unit change in the predictor, after allowing for other variables in the model. Positive numbers indicate larger eco-anxiety scores. *p < .05; **p < .01; ***p < .001

### Analysis

Analysis used SPSS 31 with the zero-inflation regression model extension. Analysis was in three stages. The first stage examined the association between eco-anxiety and the environmental activity scale. A quadratic term was included in a multiple regression to test for any evidence that activity may have declined at high levels of anxiety.

The second stage examined the distribution of the eco-anxiety scales for signs of zero inflation. Inspection of frequency distributions was followed by comparing AIC fit indices for Poisson models with or without zero inflation. The profile of those with zero anxiety scores was compared to those who recorded some levels of anxiety.

The final stage explored the factors that predicted individual differences in eco-anxiety. The SPSS routine for zero-inflated count models, based on the R pscl package zeroinfl (Zeileis et al., 2008), was used to run a series of hierarchical linear regressions using a Poisson model to test differences in means for the count part and a binomial model with a logit link to test for the probability of individuals scoring zero. Positive regression coefficients in the count analysis indicated a predictor was associated with increased eco-anxiety scores whereas positive coefficients in the binomial analysis indicated increased likelihood of scoring zero eco-anxiety. One aim was to see if religious identity or church tradition predicted either count scores or zero anxiety scores when other factors were added to the model. The same predictors were used for the count and for the binomial analyses. Predictors were added successively in the following order: Model 1, church tradition (Anglo-Catholic and Evangelical dummy variables); Model 2, personal factors (sex and age); Model 3: psychological factors (sensing, thinking, and neuroticism); Model 4: theological beliefs about creation (Dominion, Eschatology, Stewardship, and Sacramental); and Model 5, theological identity (the LIBCON scale). Initial analysis suggested that the effect of conservatism on eco-anxiety varied between traditions, so this was tested by adding an interaction effect between Evangelical and conservatism to the model and displaying this graphically.

## Results

### Eco-anxiety and Environmental Activity

The eco-anxiety and activity scales were significantly correlated with one another (*r* = .43, *df* = 3239, *p* < .001). Plotting mean activity scores against eco-anxiety score suggested that the number of eco-friendly activities increased rapidly at low levels of anxiety, but increased less rapidly thereafter (Fig. 1), but there was no evidence of a decline in activity at the highest levels of anxiety. Adding a quadratic term to the bivariate linear regression significantly improved model fit (Appendix, Table 8). As has been found elsewhere, eco-anxiety in this sample may be a motivation for environmentally friendly behaviours.

**Table 4:** Multiple regression of zero eco-anxiety symptoms

	Model 1	Model 2	Model 3	Model 4	Model 5	Model 6
Evangelical	0.856***	0.640***	0.492**	− 0.034	− 0.213	1.538**
Anglo-Catholic	0.351*	0.258	0.375*	0.710***	0.702***	0.732***
Female		− 0.999***	− 0.573***	− 0.255	− 0.221	− 0.219
Age		− 0.237***	− 0.313***	− 0.180***	− 0.177***	− 0.167***
Education		− 0.070	− 0.141*	− 0.079	− 0.060	− 0.045
Neuroticism			− 0.266***	− 0.228***	− 0.223***	− 0.222***
Thinking			0.223***	0.113*	0.095*	0.095*
Sensing			0.035	0.010	0.006	0.007
Dominion				0.169 ***	0.140***	0.140***
Eschatology				0.068**	0.048*	0.056*
Stewardship				− 0.238***	− 0.229***	− 0.231***
Sacramental				− 0.049*	− 0.035	− 0.039
Conservatism					0.212***	0.277***
EV x Con						− 0.307**

*Note*. *N* = 3240. Binomial distribution with log link. Estimates are predicted changes in the log odds ratio for recording zero eco-anxiety symptoms for a one-unit change in the predictor, after allowing for other variables in the model. Positive numbers indicate greater likelihood of recording zero eco-anxiety

*p < .05; **p < .01; ***p < .001

### Frequency Distribution of The Eco-anxiety Scale

The frequency distributions of the eco-anxiety scale showed evidence of zero inflation, with more people recording no anxiety than expected (Appendix, Fig. 4). Procedures for testing for the presence of zero inflation such as the Vuong test are not necessarily robust and no longer recommended (Wilson, 2015). However, a generalised linear model including all predictors with a Poisson distribution gave an Akaike information criterion (AIC) model fit index of 15,367.5, which was higher than the 14,593.5 obtained for fitting and equivalent zero-inflated Poisson model, showing that allowing for zero-inflation improved model fit. This suggests that accounting for zero inflation in the model was necessary, so predictors of eco-anxiety were tested separately for counts and zeros.

**Fig. 2:**
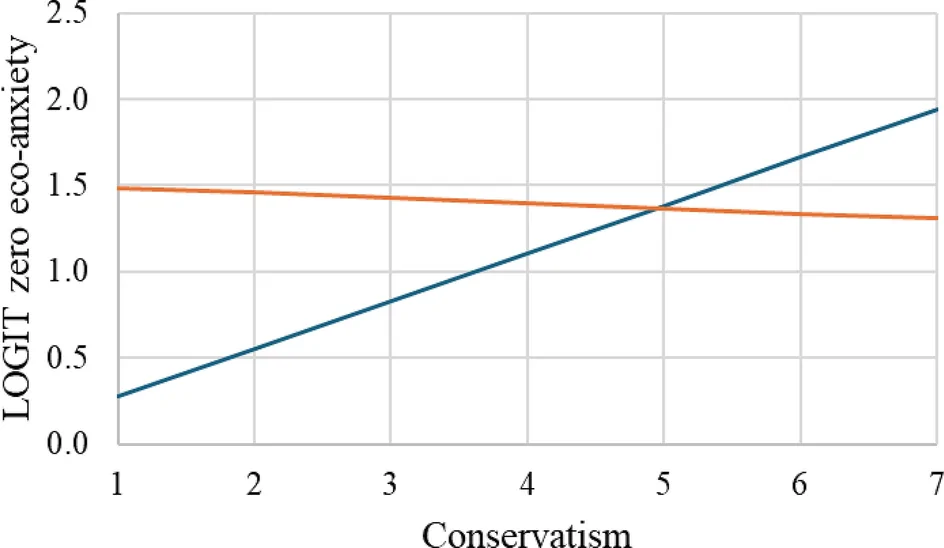
Interaction of conservatism and church tradition on eco-anxiety. *Note*: Dependent variable is the predicted change in the log odds ratio for recording zero eco-anxiety symptoms. Orange line: Evangelicals; blue line: Anglo-Catholics and Broad Church. Calculated from Model 6, Table 4

The higher-than-expected number of people reporting no eco-anxiety symptoms prompted the question of whether these represented a distinct subset of the sample. Comparison between the profiles of those with zero eco-anxiety scores and others (Table 2) showed that they were more likely to be men (71% versus 46%), more likely to be under 40 (23% versus 10%), less likely to be 60+ (40% versus 55%), and more likely to be Evangelical (42% versus 26%). There were no differences by education, but those with zero eco-anxiety had significantly lower scores for neuroticism, higher scores for thinking, and (to a lesser extent) higher scores for sensing. This suggested that those most likely to show no eco-anxiety at all tended to be young, male Evangelicals.

### Predictors of Eco-anxiety

The same predictors were used for both the Poisson count model and the zero binomial model. The estimates for the count model were the predicted changes in log symptom count for a one-unit change in the predictor, after allowing for other variables in the model (Table 3). Evangelicals were likely to report fewer anxiety symptoms than those from other traditions (Model 1), but this difference disappeared after accounting for their specific theological beliefs about creation (Model 4). Evangelicals were more likely see humans as having dominion over creation, more likely to see divine rather than human control for the fate of the Earth, less likely to view humans as stewards of creation and less likely to view creation sacramentally as imbued with the presence of God. It was these theological beliefs, alongside a conservative rather than liberal faith stance (Model 5) that tended to be associated with fewer symptoms of eco-anxiety reported by individuals. There was no evidence of an interaction effect on eco-anxiety symptom count between being Evangelical and levels of conservatism. The psychological predictors of symptom counts were as expected from previous studies in terms of increased eco-anxiety with increasing neuroticism scores. In addition, there was decreased eco-anxiety with increasing thinking scores. Sensing scores initially predicted decreased eco-anxiety (Model 3), but this was a small effect which disappeared after adding theological beliefs about creation (Model 4).

The estimates for the zero model were predicted changes in the log odds ratio for recording zero eco-anxiety symptoms for a one-unit change in the predictor, after allowing for other variables in the model (Table 4). The direction of change for increased anxiety was therefore the opposite to that of the count model. Increasing neuroticism reduced the chances of recording zero symptoms while increasing thinking scores increased the chances, which was in line with predictors in the count model and the profile of the zero-symptom group (Model 3). Sensing had no significant effect in any model. Creation beliefs and faith stance also had effects that paralleled those for the counts (Models 4 and 5): higher dominion, eschatology, and conservatism scores predicted greater likelihood of being in the zero-symptom group, while higher stewardship scores predicted the opposite. Unlike the counts regression, sacramental scores only weakly predicted being outside the zero-symptom group (Model 4), the effect disappeared when conservatism was added to the model.

The effects of church tradition on recording zero symptoms were different to the effects for predicting symptom count. In Model 1, both Evangelicals and Anglo-Catholics were more likely to record zero symptoms. This trend persisted for Anglo-Catholics when other terms were added in models 3- to 5. For Evangelicals, however, adding beliefs and conservatism in Models 4 and 5 removed the effect, suggesting that the high incidence of zero symptoms in this group was entirely a product of their theological beliefs. However, adding the interaction term for Evangelical × conservatism in Model 6 showed that conservatism had little or no effect on Evangelicals, but a strong positive effect for the other two traditions. Evangelicals, who tended to be more conservative, were more likely to be in the zero-symptom group whatever their level of conservatism, whereas for those in the Anglo-Catholic or Broad Church traditions it was only the most conservative who were likely to record zero symptoms (Fig. 2).

## Discussion and Conclusion

Anxiety over climate change and wider environmental degradation is a widespread phenomenon that is attracting increasing attention from social psychologists, not least because at low levels it may be a motivation for changing lifestyles. Whether this happens may depend on individual worldviews, including religious attitudes and beliefs. This study examined eco-anxiety in a sample of 3240 clergy and lay people from the Church of England. The Church of England embraces people with a wide range of beliefs about the environment, and whose faith varies from Catholic to Reformed and from liberal to conservative. The aim was to examine the influence on levels of eco-anxiety of religious identity or church tradition, psychological dispositions, specific theological beliefs about creation, and general liberal versus conservative stance on faith. The results lead to five main conclusions.

The first conclusion from this study is that levels of eco-friendly activity were positively correlated with levels of eco-anxiety, which parallels findings in other studies (Jain & Jain, 2022; Kricorian et al., 2025; Pavani et al., 2023; Verplanken et al., 2020; Whitmarsh et al., 2022). The relationship was not linear because there was some attenuation at higher levels of eco-anxiety, but there was no evidence that high anxiety was demotivating for eco-friendly activity, at least at the levels of anxiety recorded here. While others have suggested this might be a possibility (Wullenkord et al., 2021), the evidence is scant (Hogg et al., 2024). Whether eco-anxiety was a direct motivation for eco-friendly activity among this sample could not be determined in a cross-sectional study, but it seems a likely possibility. This means that religious attitudes and theological beliefs that reduce or prohibit anxiety about the environment might be detrimental to the desire of those churches that wish to promote care for creation. In this sense, eco-anxiety can be seen as both a symptom related to concern for the environment, but also as a motivator to do something to address the problems.

The second conclusion for this study is that eco-anxiety scores, measured as the number of ten possible symptoms, showed evidence of zero inflation, with more people recording no symptoms than expected for a typical count distribution. This is in line with the idea that some respondents may have recorded no symptoms because they were ideologically against the idea, rather than being potentially vulnerable but not showing any symptoms. This idea was supported by the profile of those with zero symptoms, who tended to be younger, male Evangelicals. It could be that this group was concerned but not anxious, rather than denying that ecological problems exist: whatever humans are doing to the environment, God is ultimately in charge, so there is no need to be anxious. Lack of anxiety here points to theological beliefs that override any psychological processes that might induce eco-anxiety. In this sample, concern over a range of environmental issues was lower among men than women, those under 50, and Evangelicals (Village & Francis, 2026), suggesting being concerned but not anxious was not a frequent response. Studies elsewhere have suggested that climate-change denial and similar attitudes towards the environment are particularly prevalent among those in Evangelical or Reformed Protestant traditions, while the growth in talk of an ecological crisis may be linked to the emergence of more liberal ideas within and beyond church circles (Defoe, 2025; Veldman et al., 2021). In these circumstances, denial may be a mixture of particular identities linked to general ideology and specific beliefs.

Third, the psychological predictors of eco-anxiety were high emotional volatility (neuroticism), low thinking scores, and to a lesser extent low sensing scores. The link between eco-anxiety and neuroticism seems to reflect how a general predisposition to anxiety or guilt can surface when thinking about the ecological crisis. It is also in line with a study of churchgoers in England that found a direct correlation between the same measure of neuroticism and concern for the environment (Village, 2020). In psychological-type models, thinking is conceived as the complementary function to feeling, which seems to be analogous to agreeableness. Thinking types tend to be more theologically and morally conservative (Village, 2019, 2024) and are more common among Evangelicals, but multiple regression suggested psychological effects were not simply due to these associations. Thinking types are characterised as tough-minded and logical, and they may be less inclined to be swayed by empathy with those who worry about the environment.

The fourth conclusion from this study is that specific theological beliefs about the environment or creation were associated with individual differences in levels of eco-anxiety. As predicted from previous studies of eco-concern in England (Village, 2015, 2020), those whose theological stance was characterised by the dominion of humans over creation, or divine rather than human control over the fate of the planet, were less likely to show symptoms of eco-anxiety whatever their psychological predispositions or tradition. Conversely, those whose theological stance was characterised by the idea of humans as stewards of creation, or the sacramental indwelling of the divine in creation, were more likely to show symptoms of eco-anxiety. Stewardship is a key idea for Christians which is not necessarily the opposite of dominion (Village, 2015). Those who think that humans are responsible to God for care of creation, irrespective of whether or not that is because humans ‘rule’ the earth, may more keenly feel anxious and guilty if things are not being cared for as they should be. If eco-anxiety is a precursor of eco-friendly activity and is itself driven by particular theological understandings of the nature of creation and the place of humans in it, then churches would seem to have a key role in ensuring that those attitudes and understandings are not supporting historical misconceptions driven by over reliance on biblical literalism (Village, 2014, 2015; White, 1967).

The fifth conclusion relates to the complex way in which faith identity and general faith stance (liberal versus conservative) interact to shape eco-anxiety in this particular population of Christians. In the Church of England, Evangelicals tend to be more conservative and Anglo-Catholics tend to be more liberal, but the effects of these two factors were slightly different depending on whether they were predicting counts of eco-anxiety symptoms or more specifically zero symptoms. For counts, Evangelical or Anglo-Catholic identity showed no effect after allowing for the specific theological beliefs associated with these different traditions. Conservatism was associated with reduced eco-anxiety across all traditions. For predicting zero symptoms (and possible ‘eco-crisis denial’), Evangelical identity predicted low eco-anxiety irrespective of levels of conservatism. Evangelicals were generally conservative, and it may have been their sense of identity, rather than their general stance on faith matters, that determined whether or not they recorded any symptoms of eco-anxiety. For Anglo-Catholics and those in the Broad Church tradition, who tended to be more liberal than Evangelicals, it was those who were most conservative who were most likely to record no eco-anxiety symptoms.

This study has shown how psychological predispositions, religious identity or church tradition, and theological beliefs can all influence eco-anxiety, sometimes in complex ways. There was evidence that some people may have rejected the notion of eco-anxiety altogether and therefore recorded no symptoms of it. Future studies might need to be sensitive to this possibility and design survey items that could better identify those who record low eco-anxiety because they reject the idea that there is any sort of ecological crisis. The fact that different theological beliefs about creation seem to promote or reduce eco-anxiety points to the importance of theological ideas, either as therapy to help alleviate extreme anxiety, or as motivations to environmentally friendly activity. At the levels of anxiety recorded in this sample, the latter would seem to be more important than the former.

These findings have implications for those who may want to find ways of reducing acute eco-anxiety among Christians. The kind of theological beliefs associated with Evangelical understandings of the relationships of God, Creation, and humans seems to offer ways of reducing anxiety without necessarily denying that problems exist. Alongside this, self-awareness related to psychological dispositions feeling over thinking or emotional volatility may help some to understand why they may be more prone to eco-anxiety than others. For churches trying to increase environmental awareness and pro-environmental behaviours, the fact that eco-anxiety is positively correlated with pro-environmental behaviours suggests there is a balance to be had between unwanted acute anxiety and lack of anxiety which might betoken indifference.

## Limitations of this Study

Although this was a large and reasonably representative sample of the Church of England it was nonetheless a convenience sample, so may not capture the views of the whole Church, especially those who attend less regularly and those who were unlikely to complete a survey. Creating a random-probability sample is difficult in the Church of England, not least because it is hard to know how different traditions are distributed across the whole Church. The *Church 2024* survey was not specifically designed to study eco-anxiety, so the items devoted to this topic were necessarily limited. Future studies that concentrate specifically on environmentalism may be able to develop more sensitive instruments. The cross-sectional nature of this study makes inferences about causality difficult, and future studies could seek to overcome this, perhaps using panel data and time-series analysis.

## Figures and Tables

**Fig. 3:**
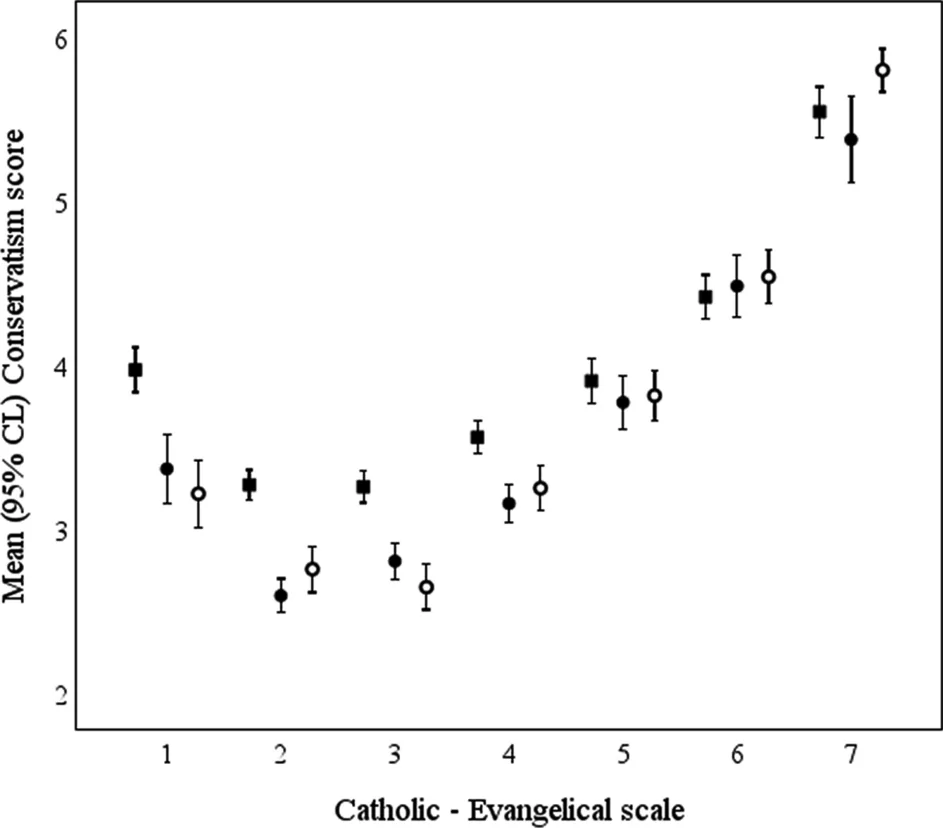
*Relationship of LIBCON scale score to Anglo-Catholic-Evangelical scale for Church Times surveys 2001 (filled squares), 2013 (filled circles), and 2024 (open circles).* Note. On the Catholic-Evangelical scale, 1–2 are Anglo-Catholic, 3–5 Broad Church, and 6–7 Evangelical. Sources of data: 2001 *Church Times* survey (Francis et al., 2005; Village, 2012b); 2013 *Church Times* survey (Village, 2018a); 2024 *Church Times* survey (present study)

**Fig. 4:**
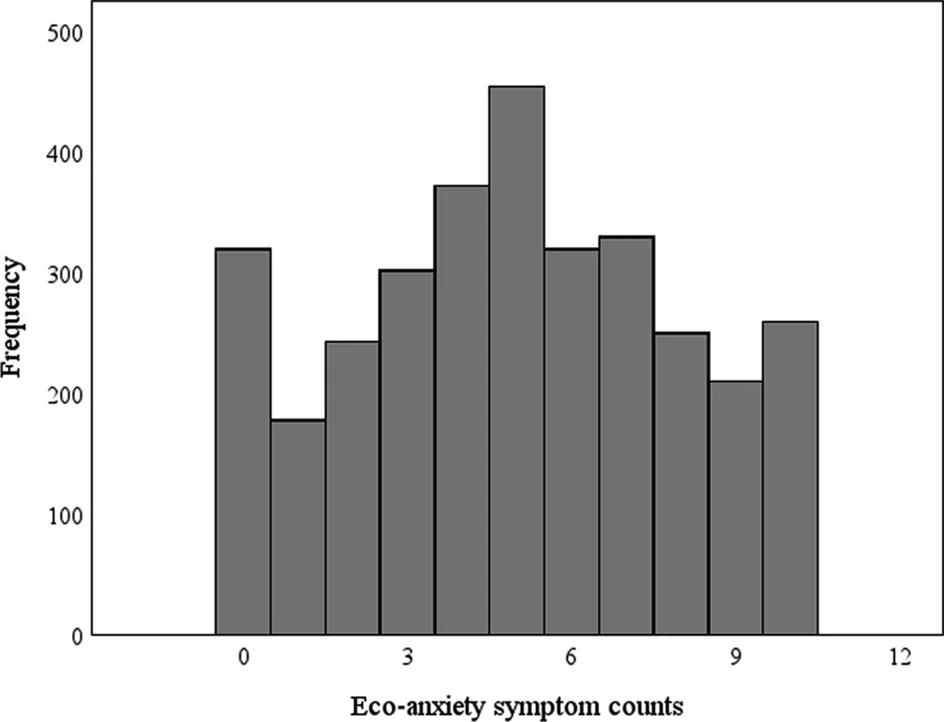
Frequency distribution of the eco-anxiety scale

## Appendix

See Tables 5, 6, 7, 8, Figs. 3, and 4.

**Table 5:** Properties of the eco-anxiety scale

Cronbach's alpha = .88; McDonald's omega = .88	Never (%)	Sometimes (%)	Most of the time (%)	Constantly (%)	CICT
Feel nervous, anxious, or on edge?	24	65	10	1	.69
Find it impossible to stop worrying?	61	35	4	1	.71
Feel afraid?	42	53	4	1	.70
Find it impossible to stop thinking about these issues?	64	32	3	1	.68
Have difficulty in sleeping?	78	21	1	0	.54
Find it hard to enjoy social gatherings?	79	19	1	0	.52
Feel guilty?	29	64	6	1	.59
Feel angry?	28	65	6	1	.60
Feel helpless?	21	60	17	3	.54
Find it hard to work or study?	78	21	1	0	.54

Note. *N* = 3240. The scale is based on the first ten items of the 13-item Hogg Eco-Anxiety Scale (Hogg et al., 2021, 2023). This included the subscales assessing affective symptoms, rumination, and behavioural symptoms, but excluded anxiety about personal impact. Items were introduced using the rubric: ‘This question is about how bothered you are by the following problems when you are thinking about climate change or other issues such as habitat loss, species extinction, pollution, waste management, or deforestation. *When you are thinking about environmental issues*, how often do you’:

*CITC* Corrected item-total correlation

**Table 6:** Properties of the eco-activity scale

Cronbach’s alpha = .76; McDonald's omega = .74	No	Yes	CITC
Have you ever:	%	%	
Given money to an environmental organisation?	35	65	.52
Actively protested about an environmental issue?	74	26	.44
Joined an environmental organisation?	66	34	.47
Changed what you eat for environmental reasons?	35	65	.49
Changed the way you travel for environmental reasons?	36	64	.47
Led a church or non-church environmental group?	85	15	.38
Attended a course about environmental issues?	61	39	.38

*Note*. *N* = 3240. *CITC* = Corrected item-total correlation

**Table 7:** Properties of the theological stance towards creation scales

	DS	D	NC	A	AS	CITC
Cronbach’s alpha = .88; McDonald's omega = .88	%	%	%	%	%	
*(a) Dominion*						
God has put human beings in charge of the earth	6	15	13	37	28	.69
The earth was created by God to supply human needs	12	35	21	25	8	.59
God wants humans to have dominion over other creatures	10	22	23	32	13	.78
Human beings are the most important species on earth	10	26	19	29	16	.70
Human beings were created to rule the earth	17	29	19	23	12	.81
God has intended the human population to expand as it has	6	21	51	16	6	.52
*(b) Eschatology*						
Cronbach’s alpha = .76; McDonald's omega = .76						
God will not allow humans to destroy the earth	8	29	45	13	6	.43
God will renew the earth 1 day	3	5	30	28	34	.53
Humans could damage the earth beyond repair*	4	10	14	39	32	.54
The future of the planet is in human hands*	6	14	19	39	22	.60
Natural forces will decide the fate of plant earth*	9	28	38	20	5	.29
*(c) Stewardship*						
Cronbach’s alpha = .80; McDonald's omega = .81						
We are stewards of creation	0	1	2	33	64	.48
Preserving Creation is one of the most important human duties	1	5	9	48	38	.36
Humans should preserve the earth because it belongs to God	1	2	8	40	49	.64
Humans are the guardians of creation	1	3	7	49	40	.57
Humans will have to account to God for their care of the earth	1	2	12	43	42	.64
*(d) Sacramental*						
Cronbach’s alpha = .83; McDonald's omega = .83						
God is revealed in Creation as much as in the Bible	3	14	9	40	33	.63
All of Creation is sacred	2	8	15	41	34	.70
God is present in all Creation	2	6	10	40	42	.69
All of Creation reveals the glory of God	1	2	6	38	53	.44
To harm the earth is to harm God	8	19	25	30	17	.60
God speaks to us through the natural world	1	2	7	52	38	.55

*Note*. *N* = 3240

*DS* disagree strongly, *D* disagree, *NC* not certain, *A* agree, *AS* agree strongly. *CICT* corrected item-total correlation. * These items were reverse coded

**Table 8:** Linear and quadratic regressions of the activity scale on the eco-anxiety scale

	Unstandardised coefficients		Standardised coefficients		
	B	SE	Beta	*t*	Sig
**(a) Linear**					
Eco-anxiety	0.29	0.01	.43	27.1	<.001
(Constant)	1.65	0.06		26.7	<.001

Model summary:	*R*	*R*^2^	Adjusted *R*^2^	SE of the estimate	
	.43	.19	.19	1.8	

	Unstandardised coefficients		Standardised coefficients		
	B	SE	Beta	*t*	Sig
**(b) Quadratic**					
Eco-anxiety	0.52	0.04	.77	14.4	<.001
Eco-anxiety**^2^	− 0.02	0.00	− .36	− 6.7	<.001
(Constant)	1.28	0.08		15.7	<.001

Model summary:	*R*	*R*^2^	Adjusted *R*^2^	SE of the estimate	
	.44	.20	.20	1.8	
